# Clinical and analytical validation of an 82-gene comprehensive genome-profiling panel for identifying and interpreting variants responsible for inherited retinal dystrophies

**DOI:** 10.1371/journal.pone.0305422

**Published:** 2024-06-13

**Authors:** Jacqueline Chan, Jolyon Holdstock, John Shovelton, James Reid, Graham Speight, Duarte Molha, Venu Pullabhatla, Stephanie Carpenter, Ezam Uddin, Takanori Washio, Hiroko Sato, Yuuki Izumi, Reiko Watanabe, Hayato Niiro, Yoshiyuki Fukushima, Naoko Ashida, Takashi Hirose, Akiko Maeda

**Affiliations:** 1 Oxford Gene Technology Operations Limited, Kidlington, Oxfordshire, United Kingdom; 2 Life Innovation Center, Riken Genesis Co. LTD, Kawasaki, Kanagawa, Japan; 3 Division of Clinical Cancer Genomics, Hokkaido University Hospital, Sapporo, Hokkaido, Japan; 4 Technology Innovation, Sysmex Corporation, Kobe, Hyogo, Japan; 5 Medical & Scientific Affairs, Sysmex Corporation, Kobe, Hyogo, Japan; 6 Department of Ophthalmology, Kobe City Eye Hospital, Kobe, Hyogo, Japan; Hebrew University Hadassah Medical School, ISRAEL

## Abstract

Inherited retinal dystrophies comprise a clinically complex and heterogenous group of diseases characterized by visual impairment due to pathogenic variants of over 300 different genes. Accurately identifying the causative gene and associated variant is crucial for the definitive diagnosis and subsequent selection of precise treatments. Consequently, well-validated genetic tests are required in the clinical practice. Here, we report the analytical and clinical validation of a next-generation sequencing targeted gene panel, the PrismGuide IRD Panel System. This system enables comprehensive genome profiling of 82 genes related to inherited retinal dystrophies. The PrismGuide IRD Panel System demonstrated 100% (*n* = 43) concordance with Sanger sequencing in detecting single-nucleotide variants, small insertions, and small deletions in the target genes and also in assessing their zygosity. It also identified copy-number loss in four out of five cases. When assessing precision, we evaluated the reproducibility of variant detection with 2,160 variants in 144 replicates and found 100% agreement in terms of single-nucleotide variants (*n* = 1,584) and small insertions and deletions (*n* = 576). Furthermore, the PrismGuide IRD Panel System generated sufficient read depth for variant calls across the purine-rich and highly repetitive open-reading frame 15 region of *RPGR* and detected all five variants tested. These results show that the PrismGuide IRD Panel System can accurately and consistently detect single-nucleotide variants and small insertions and deletions. Thus, the PrismGuide IRD Panel System could serve as useful tool that is applicable in clinical practice for identifying the causative genes based on the detection and interpretation of variants in patients with inherited retinal dystrophies and can contribute to a precise molecular diagnosis and targeted treatments.

## Introduction

Inherited retinal dystrophies (IRDs) comprise a large family of rare eye diseases characterized by the progressive dysfunction and loss of photoreceptors and retinal pigment epithelium (RPE) [1–3]. These degenerative events lead to the gradual impairment of color and night vision, peripheral visual defects, and ultimately, blindness [1, 2]. The prevalence of IRDs ranges from approximately 1 in 2,000 to 1 in 3,000 individuals [4, 5] and is thought to involve nearly 300 different genes, each of which plays a crucial role in the survival, function, and metabolism of retinal cells [3]. Because these genes are encoded in the nuclear genome or mitochondrial DNA, their inheritance patterns can be autosomal-recessive, autosomal-dominant, X-linked, or mitochondrial [6, 7].

IRDs include retinitis pigmentosa (RP) which is the most frequently observed IRD in clinical practice, cone dystrophy (CD), and Leber congenital amaurosis (LCA) which is a child onset retinal dystrophy [8, 9]. Precise clinical diagnosis of RP, CD and LCA, along with other IRDs, is crucial in determining the etiopathogenesis of IRDs and in selecting effective treatments. However, their clinical diagnosis is complicated owing to broad clinical symptoms resulting from diverse pathogenic variants of different causative genes [9–11]. IRDs are highly heterogeneous both in terms of phenotype and genotype [12–14]. Therefore, an accurate molecular diagnosis is essential for definitively diagnosing IRDs.

Genetic testing using next-generation sequencing (NGS) has considerably advanced the molecular diagnosis of IRDs by enabling the identification of causative genes [15–17]. As a result, determining the genotype of an IRD is increasingly considered an essential component for establishing a definitive diagnosis and selecting patients eligible for gene therapies [18, 19]. The Food and Drug Administration has approved the first gene therapy for patients with LCA caused by biallelic variants in *RPE65*. This gene therapy, voretigene neparvovec-rzx (Luxturna), delivers a functional copy of *RPE65* to RPE cells in patients using an adeno-associated virus vector and has achieved notable improvements in visual function [20–22]. This achievement in gene therapy has stimulated the further exploration of other candidate genes [23]. Multiple phase 3 clinical trials are currently underway for gene therapies targeting *CEP290* for LCA, *CHM* for choroideremia, and *RPGR* for X-linked RP [24, 25]. These emerging gene therapies underscore the importance of genetic testing to provide precise molecular information and to guide the most beneficial gene therapies.

Currently, many NGS-based genetic tests utilize short-read NGS because of its ability to deliver large volumes of highly reliable sequence data at relatively low costs. However, variant detection in repetitive sequences remains challenging in short-read sequencing [26]. This limitation complicates the sequencing of the open reading frame 15 (ORF15) of *RPGR*, which is implicated in X-linked RP and has a hot spot for disease-causing variants in a highly purine-rich and repetitive region [27–30]. Therefore, due to this limitation associated with short-read NGS, a precise molecular diagnosis of *RPGR* ORF15 currently still often relies on traditional Sanger sequencing in clinical practice.

In addition, accurate interpretation of variants is crucial for providing effective care for patients with IRDs. The American College of Medical Genetics and Genomics (ACMG), together with the Association for Molecular Pathology (AMP), has reported guidelines and standards for interpreting variants in a systematic and structural manner [31]. Although the ACMG/AMP guidelines have increased the consistency of variant interpretation, they remain complex.

In this study, we aimed to develop an NGS-based targeted gene panel and software (the PrismGuide IRD Panel System) that was designed to identify the causative 82 genes of IRDs. This system has now received regulatory approval from the Ministry of Health, Labor, and Welfare in Japan (Approval number: 30500BZX00129000, Manufacturer: Sysmex Corporation). Analytical and clinical validation of the PrismGuide IRD Panel System demonstrated that it can accurately and reproducibly detect variants in 82 genes related to IRDs and automatically interpret the pathogenicity of variants based on the ACMG/AMP guidelines. The results of our study suggest that the PrismGuide IRD Panel System is useful for precise molecular diagnosis, especially for specific decision-making in clinical practice.

## Materials and methods

### Reference-genome DNA and clinical samples

A well-characterized and commonly used human male reference genome, NA24385, was obtained from Coriell Institute. A cohort of Japanese patients with IRDs was recruited from an IRD clinic in Kobe City Eye Hospital, the Institute of Biomedical Research and Innovation Hospital [32]. Peripheral whole blood was collected from patients with IRDs into conventional ethylenediaminetetraacetic acid (EDTA) blood-collection tubes. The collected blood was either used immediately for DNA extraction or stored at -20°C for subsequent use.

The research protocols in this study were approved by the institutional review boards of Kobe City Medical Center General Hospital (Research number: E19002 and E20010) and Sysmex Corporation (Registration number: 2020–243 and 2021–077). In E20010, patients with IRDs were recruited from October 2021 through September 2022, and written informed consent was obtained. In E19002 and 2020–243, the data and archived samples were accessed after 10 November 2020. In 2021–077 the data and archived samples were accessed from January 2022.

The cohort comprised 47 patient with IRD patients who were clinically diagnosed with Bietti crystalline retinopathy (*n* = 1), choroideremia (*n* = 1), cone-rod dystrophy (*n* = 2), Leber congenital amaurosis (*n* = 2), macular dystrophy (*n* = 2), retinitis pigmentosa (*n* = 36), retinoschisis (*n* = 1), and Usher syndrome (*n* = 1), and a parent of a proband with retinitis pigmentosa (*n* = 1) (S2 Table). This cohort was used to determine the accuracy of variant detection for SNV, small indel and CNV-loss.

### Characteristics and assay workflow of the PrismGuide IRD Panel System

The PrismGuide IRD Panel System, which is primarily developed by Oxford Gene Technology Operations Limited (United Kingdom), is a comprehensive genome-profiling panel that targets 82 genes related to IRDs obtained from literature reviews and the Retinal Information Network (RetNet) (https://web.sph.uth.edu/RetNet/) (Table 1). These 82 genes were selected based on the following four criteria: 1) a gene, *RPE65*, that is a target of the available gene therapy, voretigene neparvovec-rzx (Luxturna), 2) 23 genes that are the targets of prospective therapies which are under clinical trials or investigational studies, 3) 34 genes associated with two or more pedigrees of the Japanese population with IRDs, and 4) 24 genes frequently associated with IRDs in other populations in addition to the Japanese population. The 82 genes are associated with retinitis pigmentosa (71%, 58 genes), cone or cone-rod dystrophy (29%, 24 genes), Leber congenital amaurosis (18%, 15 genes), macular degeneration (11%, 9 genes), and other genetic retinal diseases accounting for a small proportion in the panel. In RetNet, some genes are associated with multiple IRD-related diseases. The RefSeq transcripts (GRCh38) of these 82 genes are provided in S3 Table.

**Table 1:** List of genes targeted in the PrismGuide IRD Panel System. The target regions include all exons of 82 genes and an intron between exon 26 and 27 in *CEP290*, where pathogenic variants are observed [33].

*ABCA4*	*CNGA1*	*GUCA1A*	*NRL*	*PRPH2*	*RPGR*
*ADGRV1*	*CNGA3*	*GUCY2D*	*NYX*	*RBP3*	*RPGRIP1*
*AIPL1*	*CNGB1*	*IDH3B*	*PCARE*	*RDH12*	*RS1*
*BEST1*	*CNGB3*	*IMPDH1*	*PDE6A*	*RDH5*	*SAG*
*C8orf37*	*CRB1*	*IMPG2*	*PDE6B*	*RGR*	*SEMA4A*
*CA4*	*CRX*	*IQCB1*	*PDE6C*	*RGS9BP*	*SNRNP200*
*CACNA1F*	*CYP4V2*	*KCNV2*	*PDE6G*	*RHO*	*SPATA7*
*CDH23*	*DHDDS*	*KLHL7*	*POC1B*	*RLBP1*	*TOPORS*
*CDHR1*	*DRAM2*	*LRAT*	*PRCD*	*ROM1*	*TTC8*
*CEP290*	*EYS*	*MAK*	*PROM1*	*RP1*	*TULP1*
*CERKL*	*FAM161A*	*MERTK*	*PRPF3*	*RP1L1*	*USH2A*
*CFAP410*	*FSCN2*	*MYO7A*	*PRPF31*	*RP2*	*ZNF513*
*CHM*	*GNAT2*	*NMNAT1*	*PRPF6*	*RP9*	
*CLRN1*	*GRK1*	*NR2E3*	*PRPF8*	*RPE65*	

**Fig 1:**
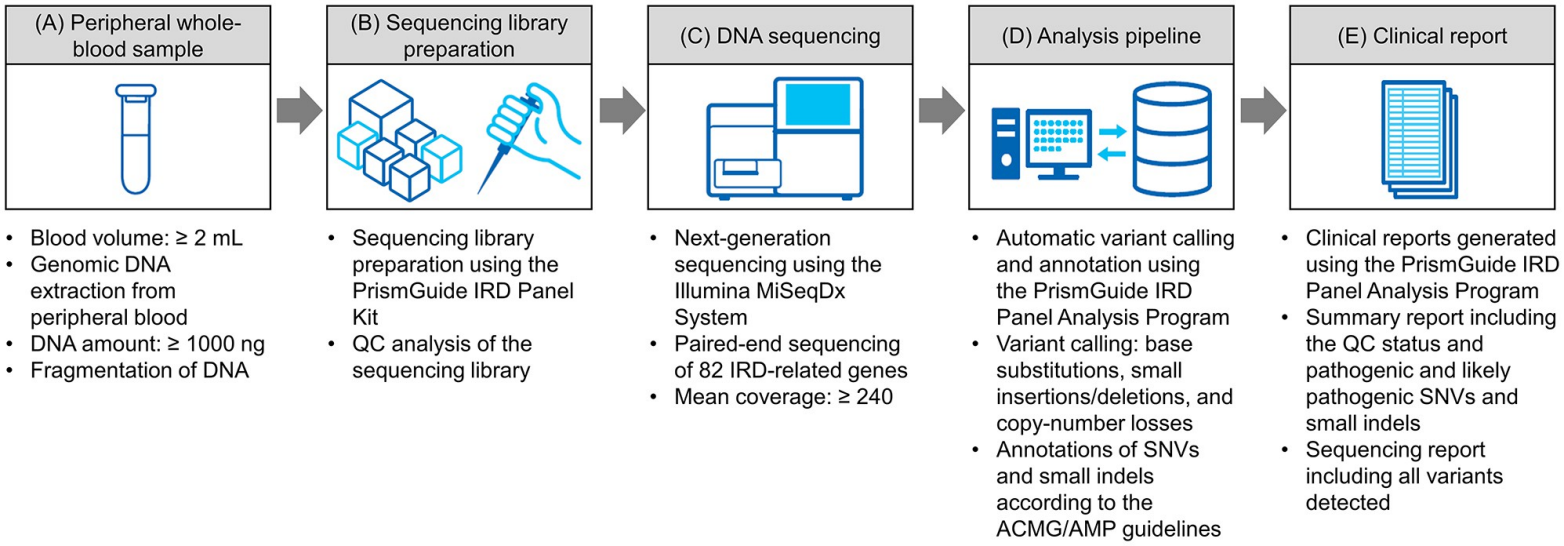
Workflow used with the PrismGuide IRD Panel System for genomic profiling. (A) Genomic DNA was extracted from routine peripheral whole-blood samples. (B) Sequencing libraries were prepared using the PrismGuide IRD Panel Kit with ≥1000 ng of genomic DNA, and 82 genes related to IRDs were enriched by hybrid capture. The quality of each sequencing library was evaluated by DNA quantification. (C) DNA sequencing was performed using the Food and Drug Administration-regulated Illumina MiSeqDx System. (D) Germline variants for single-nucleotide variants (SNVs), small insertions and deletions (indels), and copy-number loss were called using the PrismGuide IRD Panel Analysis Program. The SNVs and small indels were annotated in terms of their pathogenicity according to the ACMG/AMP guidelines by the PrismGuide IRD Panel Analysis Program [31]. (E) Clinical reports were generated using the PrismGuide IRD Panel Analysis Program. The "Summary report” included the status of QC analysis and pathogenic and likely pathogenic SNV and small indels, classified using the five-tier system mentioned in the ACMG/AMP guidelines [31]. The “Sequencing report” included all variants detected and information pertaining to the analysis program and databases used.

These genes serve as therapeutically relevant biomarkers for approved gene therapy, enrollment in clinical trials, visual-prognosis assessments, and potential risk assessments for family members. The workflow used with the PrismGuide IRD Panel System is illustrated in Fig 1. Briefly, at least 2 mL of peripheral whole blood was drawn from patients with clinically diagnosed or suspected IRDs, and genomic DNA was extracted from 200 μL of blood using the QIAamp DNA Blood Mini Kit (Qiagen, Germany) according to the manufacturer’s instructions. Sequencing library preparation was performed using the PrismGuide IRD Panel Kit with a minimum input of 1,000 ng DNA. The DNA was fragmented using a Covaris focused-ultrasonicator (Covaris, LLC, Woburn, MA) and end-repaired to generate blunt-ended DNA with free 5′ phosphate groups prior to ligating adapters necessary for downstream sequencing. Adaptor-ligated DNA was amplified by polymerase chain reaction (PCR) using specific index primers to add index sequences to enable sample identification and multiplexing. The samples were then hybridized under controlled conditions to baits specifically designed using proprietary algorithms to enable the capture and enrichment of the targeted DNA regions. The enriched libraries were subsequently amplified by PCR, and the products were quantified by Qubit (Thermo Fisher Scientific, Waltham, MA) for quality control (QC) assessment.

The libraries were sequenced using the Food and Drug Administration-regulated MiSeqDx system (Illumina, Inc. San Diego, CA) and paired-end sequencing. The acquired sequencing reads were analyzed using the PrismGuide IRD Panel Analysis Program to detect single-nucleotide variants (SNVs), small insertions and deletions (indels), and copy-number loss. The pathogenicity of the detected variants was evaluated according to the ACMG/AMP guidelines [31]. The PrismGuide IRD Panel Analysis Program was used to generate two clinical reports. One report was the “Summary report” that included the pathogenic and likely pathogenic variants identified along with the QC status. The other report was the “Sequencing report” that included all variants detected.

### Bioinformatics analysis for detecting variants and evaluating variant pathogenicity

#### Data processing

The data generated with the PrismGuide IRD Panel System were analyzed using a pipeline derived from open-source tools in combination with custom-written, in-house algorithms. The analysis pipeline included FASTQ sequence data, a BED file defining the IRD-panel target regions, and a configuration file detailing the information for individual samples.

Genome alignment was performed using the BWA-MEM algorithm with GRCh38 as the reference genome. Aligned read files were sorted, and duplicates were marked using the Sambamba filter to generate an alignment-map file (BAM) and an index (BAI). Following alignment, the read depths across the alignments were calculated to generate per-base read depths, and the high-quality filtered BAM output file was used for variant detection.

#### Variant detection and evaluation

Copy-number loss events were detected by comparing the normalized fold depth of coverage using the proprietary pipeline. Reads from the sample alignment were used to derive the mean depth of coverage across a binned version of the IRD panel. The binned version of the IRD panel is the panel target regions divided into consecutive segments, each of length 50. A copy-number loss was reported if more than three consecutive bins were significantly lower than the depth of coverage in the bins spanning the same region in the pool of reference samples. This setting implies a resolution of copy-number loss calling of 150 base pairs.

SNV and small indel calling was performed using the Freebayes software, and the identified variants were normalized and stored in a VCF file. The variants listed in the VCF file were annotated according to independently curated data and publicly available resources. Subsequently, the annotated variants were classified following the standards and guidelines for interpreting sequence variants recommended by the ACMG/AMP guidelines, which were implemented in the Prism Guide IRD Panel System software. The genotype of a variant is also predicted by the software based on the allele frequency.

#### QC analysis

QC analyses of the raw sequencing reads were performed using fastp to generate a large set of metrics and statistics. The selected metrics included Q20 and Q30 base scores, guanine and cytosine (GC) contents, insertion size estimations, the presence of adapters, the base quality, and the base content. A second QC of the read alignment was used to provide additional metrics related to coverage efficiency, areas not covered, and estimated insertion sizes.

### Accuracy of SNV and small indel calling

For the accuracy study, we selected 43 variants comprising 35 SNVs and eight small indels from 42 patients with IRDs. These variants were previously characterized using targeted gene-panel sequencing or Sanger sequencing. The accuracy of the PrismGuide IRD Panel System, compared with that of Sanger sequencing, was tested by determining the concordances for all 43 variants detected. Bidirectional Sanger sequencing was independently developed as an orthogonal method to confirm these 43 variants. We performed PCR with gene-specific primers to amplify the targeted genomic regions using 30 ng of DNA. The PCR products were purified using the ExoSAP-IT PCR Product Clean Up Kit (Thermo Fisher Scientific, Waltham, MA) and were used for sequencing reaction with the BigDye Terminator 3.1 Cycle Sequencing Kit (Thermo Fisher Scientific, Waltham, MA), followed by DNA sequencing using the Applied Biosystems 3730xl DNA analyzer (Thermo Fisher Scientific, Waltham, MA) with M13 forward and reverse primers. SeqScape Software (Thermo Fisher Scientific, Waltham, MA) was used to identify all 43 targeted variants, and the results were confirmed by visual inspection. Detection of these 43 variants was assessed using the PrismGuide IRD Panel System to determine the accuracy of the PrismGuide IRD Panel System compared to orthogonal Sanger sequencing for variant detection.

For the SNVs and small indels, a result was counted as a true positive (TP) if the PrismGuide IRD Panel System detected a variant identical to that identified by Sanger sequencing. A false-positive (FP) result was defined as an instance where the PrismGuide IRD Panel System detected a variant that was not identified by Sanger sequencing. A false-negative (FN) result was defined as an instance where the PrismGuide IRD Panel System did not detect a variant that was identified by Sanger sequencing, although it satisfied the QC metrics in the PrismGuide IRD Panel System. A true-negative (TN) result was defined when a given variant was not identified with both the PrismGuide IRD Panel System and Sanger sequencing in the targeted genomic region despite satisfying QC metrics in both assays. The accuracy was determined by the positive-percent agreement (PPA; defined as TP/[TP + FN] and the negative-percent agreement (NPA; defined as TN/[TN + FP]) [34]. The associated 95% confidence interval (CI) was determined using the Clopper–Pearson method.

### Accuracy of detecting copy-number loss

We selected five unique copy-number losses from five patients whose genomes were previously characterized by whole-genome sequencing. This enabled us to estimate the genomic locations of the breakpoints for these copy-number losses. To confirm these five copy-number losses, we developed a validated orthogonal PCR method. Briefly, two PCRs were performed with different primer sets. One primer set flanked the deletion breakpoints to detect the deletion allele, and the other primer set was designed to amplify a region inside the deletion allele to detect the wild-type allele. Therefore, homozygotes for copy-number loss only showed one band of the deletion allele on an agarose gel following PCR, heterozygotes for copy-number loss showed two bands (deletion and wild-type alleles), and wild-type individuals only showed one band of the wild-type allele. The accuracy of the PrismGuide IRD Panel System in copy-number loss detection was determined by assessing the concordance between the detection of copy-number loss with the PrismGuide IRD Panel System and with the PCR approach.

### Precision of detecting SNVs and small indels

The repeatability and reproducibility of variant detection were evaluated by detecting 15 unique variants in a well-characterized human male reference genome (NA24385; S1 Table). These variants included 11 SNVs located in exons or introns of five genes and four small indels in four genes (ranging from 1 to 22 base pairs). To assess the precision, two different operators used three different reagent lots, where each lot was prepared for sequencing libraries over three independent days. Sequencing libraries were prepared using eight NA24385 samples each day, resulting in 144 replicates of NA24385 samples representing 2,160 variants, including 1,584 SNVs and 576 small indels. The variants were detected in multiple sequencing runs. Repeatability was assessed by comparing eight replicates tested by the same operator on the same day with the same reagent lot. Reproducibility was assessed by two operators over three days using three different reagent lots.

### Robustness of SNV and small indel detection in the presence of potentially interfering substances and different storage conditions

To assess the impact of potential endogenous and exogenous interfering substances on variant detection, peripheral whole blood was collected from three patients with IRDs and tested under 10 different conditions. The effects of endogenous interfering substances were tested by adding either 137 μM or 684 μM bilirubin conjugate, 6.2 μM or 31 μM hemoglobin, 2.6 μM or 13 mM cholesterol, or 7.4 mM or 37 mM triglycerides to the blood. The effects of the exogenous interfering substance were assessed in the presence of EDTA at a concentration of 9.5 μM or 23 μM in the blood. The concentration of the interfering substances was obtained based on the recommended concentration specified in the Clinical and Laboratory Standards Institute (CLSI) guidelines or the document review of another NGS genetic test that is regulatory-approved by the Food and Drug Administration.

DNA was extracted from the blood and used to prepare sequencing libraries using the PrismGuide IRD Panel Kit. Subsequently, DNA sequencing was performed, followed by variant detection using the PrismGuide IRD Panel Analysis Program. To determine the rate of variant detection, the number of variants detected in the presence of interfering substances was compared with that observed without adding interfering substances.

Peripheral whole blood and extracted DNA were stored under five different conditions to test the impact of storage conditions on the sample quality. Peripheral whole blood was drawn from 11 patients with IRDs, and aliquots of each blood were stored at 4°C for 30 days or 30°C for 7 days. Extracted DNA was stored at 4°C for 30 days, -20°C for 60 days, or -20°C for 90 days. These conditions were determined based on the prospective operation process of the PrismGuide IRD Panel System. These high temperatures and long-term storage are unfavorable for the stability of whole blood and DNA samples and could potentially interfere with the NGS assay. After storage under these conditions, the PrismGuide IRD Panel System was used to detect the variants. To determine the rate of variant detection, the number of variants detected under each storage condition was compared with that observed in a control condition, in which peripheral whole blood and DNA were immediately processed for library preparation without an extended storage period or unusual temperature conditions.

## Results

### Accuracy of SNV and small indel detection

**Fig 2:**
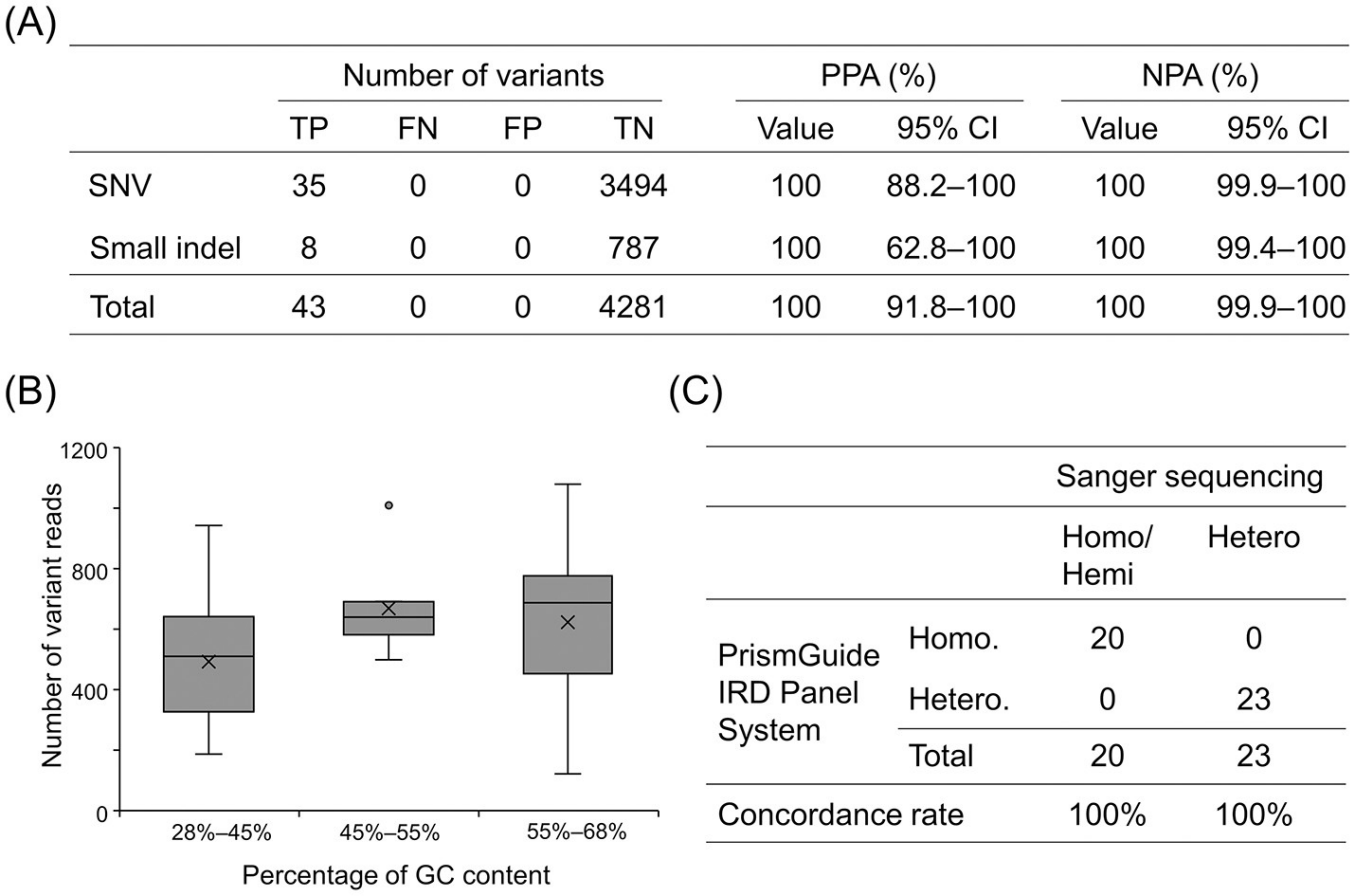
Accuracy of variant detection with PrismGuide IRD Panel System, compared to Sanger sequencing. (A) The TP, FN, FP, TN, PPA, and NPA found for the variants of interest are indicated. SNV, single-nucleotide variant; indel, insertion and deletion; TP, true positive; FN, false negative; FP, false positive; TN, true negative; PPA, positive-percent agreement; NPA, negative-percent agreement; CI, confidence interval. (B) Number of variant reads with the indicated GC-content levels is shown in the boxplot. Each cross represents the mean of the reads. (C) The zygosity of the variants determined with the PrismGuide IRD Panel System was compared with that observed via Sanger sequencing. The numbers of homozygous/hemizygous and heterozygous variants and concordance rate are indicated in the two-by-two contingency table. Homo, homozygote; Hemi, hemizygote; hetero, heterozygote.

Variant detection using the PrismGuide IRD Panel System was compared with that of the orthogonal Sanger sequencing method using peripheral whole blood obtained from 42 patients with IRDs. Using samples from these patients, Sanger sequencing identified 35 SNVs in 24 genes and eight small indels in seven genes, consisting of a 1-base insertion, six unique deletions with one or two bases, and one complex indel with a 2-base insertion and a 17-base deletion. The PrismGuide IRD Panel System detected all 35 SNVs and eight small indels, indicating that the PPA was 100% (95% CI: 91.8%–100%; Fig 2A). To derive the NPA, 4,281 bases harboring the identified 43 SNVs and small indels were confidently determined by Sanger sequencing, and these bases were compared with those determined with the PrismGuide IRD Panel System. All bases were concordant between both methods, indicating that the NPA was 100% (95% CI: 99.9%–100%; Fig 2A).

A GC-content bias typically exhibits a unimodal distribution in terms of read depth in hybrid capture-based NGS, where the read depth generally decreases at a high or low GC content, resulting in a lower detection rate [35–37]. Therefore, we investigated whether the GC content affected the read depth of the 43 variants. The GC content was defined as 200 bases surrounding each variant and ranged from 28% to 68%. The PrismGuide IRD Panel System showed mean read depths of 494 (*n* = 22), 669 (*n* = 8), and 623 (*n* = 13) at variant positions located in regions with GC contents ranging from 28% to 45%, 45% to 55%, and 55% to 68%, respectively (Fig 2B). These results indicate that the PrismGuide IRD Panel System can obtain sufficient read depth for variant detection over a GC-content range of 28% to 68%.

Because the zygosity of a variant is crucial in clinical practice for identifying causative genes, the PrismGuide IRD Panel System was designed to report the zygosity of variants. To evaluate its accuracy, the zygosity determined using the PrismGuide IRD Panel System was compared with that determined using Sanger sequencing for all 43 variants. We found that both Sanger sequencing and the PrismGuide IRD Panel System concordantly identified 20 homozygous/hemizygous variants and 23 heterozygous variants, indicating that the concordance rate was 100% (Fig 2C). The variant allele frequency (VAF) was 92.6%–100% in homozygotes (which theoretically reflects a 100% VAF), and it was 40.6%–55.3% in heterozygotes (which theoretically represents a 50% VAF). Based on these results, we concluded that the PrismGuide IRD Panel System accurately identified the zygosity of the variants.

### Accuracy of copy-number loss detection

To assess the accuracy of copy-number loss detection, first whole-genome sequencing was utilized to identify large deletions and estimate their breaking points in the genome in five patients with IRDs (S884, S952, S983, S1042, and S1145). Five unique deletions in four genes (*RP1L1*, *PDE6B*, *EYS*, and *PRPF31*) with deletion sizes ranging from 3.9 kb to 93.2 kb were identified. All deletions were located in regions targeted by the PrismGuide IRD Panel System. Subsequently, these deletions were confirmed using orthogonal PCR analysis to distinguish the genotypes among the wild-type, heterozygous-deletion, and homozygous-deletion alleles. PCR analysis revealed that all five deletions were heterozygous, indicating a copy number of one (Table 2). The accuracy of copy-number loss was assessed using the PrismGuide IRD Panel System, showing that the PrismGuide IRD Panel System detected four of five deletions (Table 2). Collectively, the copy number for these deletions was also determined as one (range of copy numbers: 1.01–1.11), consistent with the results obtained from the orthogonal PCR analysis. These results showed that the PrismGuide IRD Panel System had a positive agreement of 80% (*n* = 5) in detecting copy-number loss and, at least mostly, was capable of determining the copy number correctly.

**Table 2:** Accuracy of copy-number loss detection.

Patient ID	Gene	Exon^#1^	PCR		PrismGuide IRD Panel System	
			Copy-number loss	Copy number	Copy-number loss	Copy number
S884	*RP1L1*	3–4	Detected	1	Detected	1
S952	*PDE6B*	2–3	Detected	1	Not detected	n/a^#2^
S983	*EYS*	6–8	Detected	1	Detected	1
S1042	*PRPF31*	1–14	Detected	1	Detected	1
S1145	*PRPF31*	2–3	Detected	1	Detected	1

^#1^ The numbers of deleted exons are indicated.

^#2^ n/a: not applicable

### Precision of SNV and small indel detection

**Fig 3:**
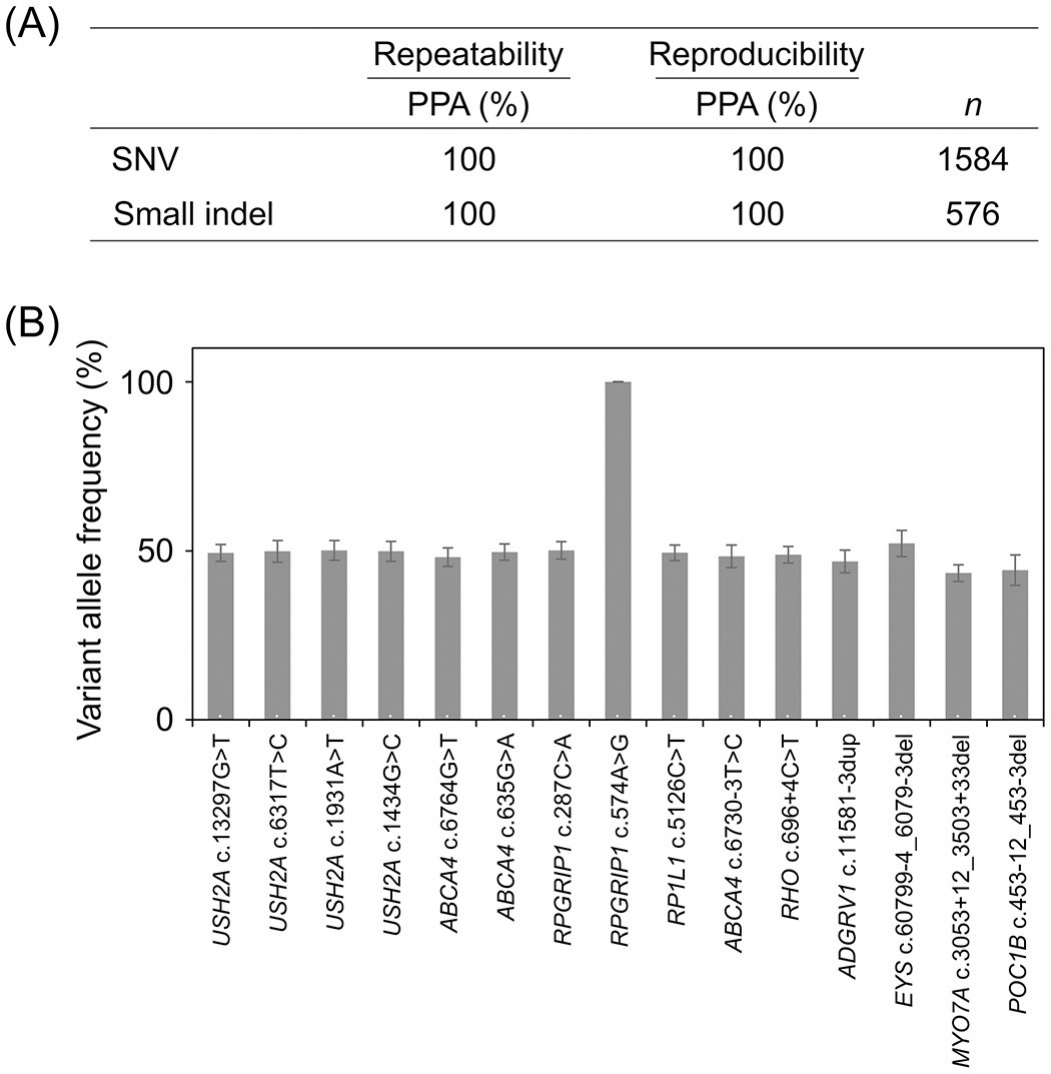
Precision of variant detection with the PrismGuide IRD Panel System. (A) The PPA values for detecting SNV and small indels with the PrismGuide IRD Panel System are shown with respect to repeatability and reproducibility. Percentages of agreement of 1,584 SNVs and 576 small indels in 144 replicates of NA24385 samples were indicated. SNV, single-nucleotide variant; indel, insertion and deletion; PPA, positive-percent agreement; *n*, Total number of SNVs or small indels tested in 144 replicates. (B) The graph shows the variant allele frequencies of the indicated variants. Error bars, standard deviation of 144 replicates.

To determine the repeatability and reproducibility of variant detection, we used the human reference genome, NA24385, which has 15 representative variants, including 11 SNVs in five genes (*ABCA4*, *RHO*, *RP1L1*, *RPGRIP1*, and *USH2A*) and four small indels ranging from 1 to 22 base pairs in four genes (*ADGRV1*, *EYS*, *MYO7A*, and *POC1B*), all of which were covered by the PrismGuide IRD Panel System. The PrismGuide IRD Panel System was employed to detect these 15 variants with two different operators on three independent days using three different reagent lots with eight replicates each, resulting in 144 replicates. The repeatability of variant detection was assessed with eight replicates under the same conditions and showed 100% agreement for the SNV (*n* = 1,584) and small indels (*n* = 576), as shown in Fig 3A. Reproducibility was also evaluated with the 144 replicates under different conditions, on different days, and with different reagent lots and operators. The results showed 100% agreement for the SNVs and small indels (Fig 3A). These results indicate that the PPA for repeatability and reproducibility was 100% both for SNVs (*n* = 1584) and small indels (*n* = 576).

Next, variations in the VAFs of the 15 representative variants were evaluated. In heterozygous variants, the mean VAF in the 144 replicates ranged from 43.4% (*n* = 144) for *MYO7A* (NM_000260.4: c.3053+12_3053+33del) to 52.2% (*n* = 144) for *EYS* (NM_001292009.2: c.6079-4_6079-3del), close to the theoretical VAF of 50.0% for heterozygous alleles. The standard deviations of the VAFs ranged from 2.3 for *RP1L1* (NM_178857.6: c.5126C>T) to 4.5 for *POC1B* (NM_172240.3: c.453-12_453-3del). In contrast, the mean VAF for the homozygous *RPGRIP1* variant (NM_020366.4: c.574A>G) was 100% (*n* = 144), identical to the theoretical VAF for homozygous alleles. In addition, the standard deviation of the VAF was 0.1. These results indicate that the VAF variations were low enough to reproducibly determine the VAF and correctly identify the zygosity of the variants.

### Accuracy of *RPGR* ORF15 detection

**Fig 4:**
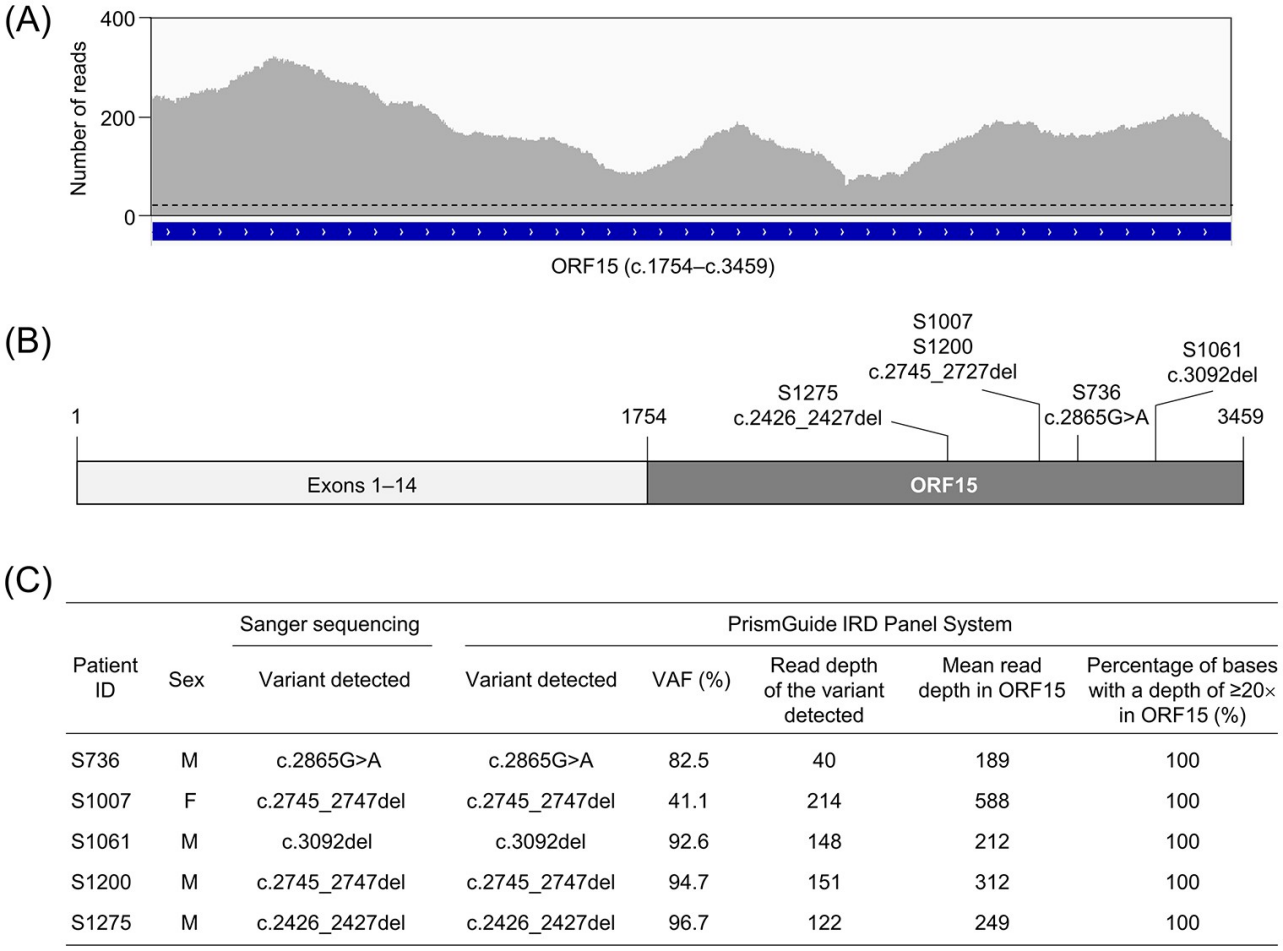
Accuracy of the detection of *RPGR* ORF15 variants with the PrismGuide IRD Panel System. (A) Reads generated with the PrismGuide IRD Panel System for *RPGR* ORF15 (NM_001034853.2: c.1754–c.3459) in one patient, S736, are indicated by the gray peaks. The genomic organization of *RPGR* ORF15 is shown in the blue box. The dashed line indicates a 20× read depth. (B) Schematic representation of the transcript of a splice isoform of *RPGR* ORF15. Exons 1–14 and ORF15 are indicated by the light and dark gray boxes, respectively. The ORF15 variants from five patients with IRDs are also indicated. (C) Accuracy of variant detection with the PrismGuide IRD Panel System, compared with that of Sanger sequencing. The variants detected, variant allele frequency, read depth of the variant, and mean read depth in the ORF15 region (NM_001034853.2: c.1754–c.3459) are indicated for the PrismGuide IRD Panel System. The percentage of bases with a depth of ≥20× in ORF 15 is also shown. M, male; F, female; VAF, variant allele frequency.

The *RPGR* ORF15 isoform includes exons 1 to 14 and ORF15, which consists of exon 15 and extends into intron 15 [38]. ORF15 encompasses c.1754–c.3459 of the transcript NM_001034853.2 and contains highly repetitive and purine-rich regions that are difficult to sequence using short-read sequencing technologies and are prone to errors [28, 39, 40]. Given this challenge, the PrismGuide IRD Panel System was improved using a proprietary hybrid-capture bait design to enhance the detection of ORF15 variants. First, to evaluate the read depth in ORF15, the PrismGuide IRD Panel System was tested using peripheral whole blood from five patients with IRDs, all of whom harbored an ORF15 variant. The mean read depth of the ORF15 region in a female (S1007) was 588 and ranged from 189 to 312 in males (S736, S1061, S1200, and S1275) (Fig 4C). Furthermore, throughout the ORF15 region, the read depth was more than 20×, which was required for variant detection in the PrismGuide IRD Panel System (Fig 4A and 4C). These results show that the PrismGuide IRD Panel System obtained a sufficient read depth to call ORF15 variants.

To assess the ORF15 variant-detection ability, five small variants from five patients with IRDs were identified by orthogonal Sanger sequencing. These variants included one SNV and four small deletions: c.2865G>A in S736, c2745_2747del in S1007, c.3092del in S1061, c.2745_c.2747del in S1200, and c.2426_2427del in S1275 (Fig 4B and 4C). We found that the PrismGuide IRD Panel System detected these variants with read depths ranging from 40 to 151 in the male and 214 in the female sample (Fig 4C). The allele frequencies of these variants ranged from 82.5% to 96.7% in the male sample and was 41.1% in the female sample. These values were close to the theoretical VAF of 100% for hemizygotes and 50% for heterozygotes. These results, therefore, suggest that the PrismGuide IRD Panel System can detect ORF15 variants and identify their zygosity correctly.

### Robustness of variant detection in clinical samples in the presence of interfering substances and different sample storage conditions

**Fig 5:**
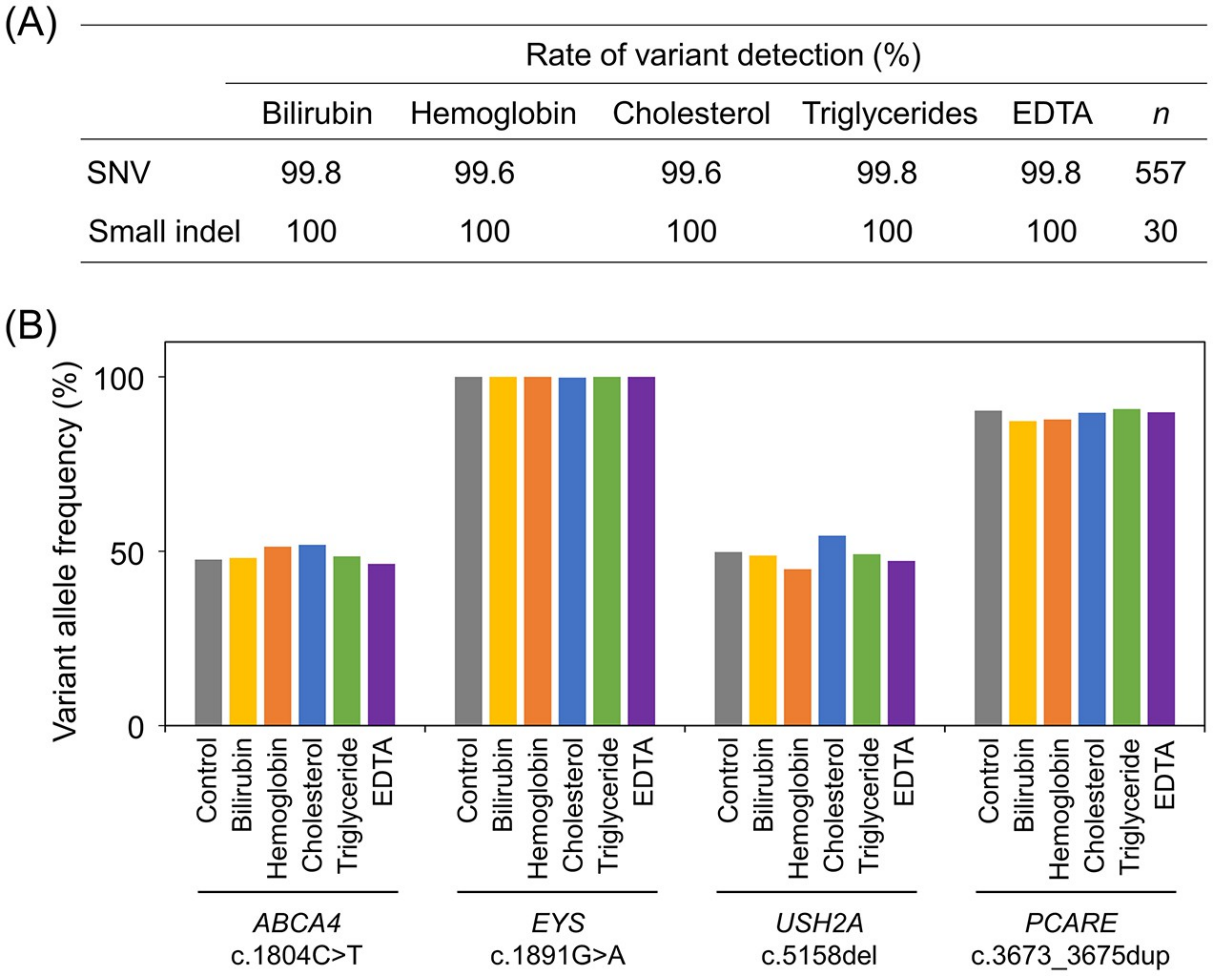
Effect of interfering substances on variant detection in the PrismGuide IRD Panel System. (A) The percentages of variant detection in the presence of the indicated interfering substances were shown. The percentage of variant detection was determined by comparing the number of variants detected in the presence of the interfering substances in three clinical samples with that found in the control condition, where no interfering substances were added. The numbers of the variants detected are shown in parentheses. SNV, single-nucleotide variant; indel, insertion and deletion; EDTA, ethylenediaminetetraacetic acid. (B) Variant allele frequencies of the representative variants in the presence of the interfering substances are shown. The control condition involved sequencing in the absence of the interfering substances.

Because peripheral whole blood contains substances that can interfere with variant detection via NGS, the effects of potential endogenous interfering substances were tested, including bilirubin conjugates, hemoglobin, cholesterol, and triglycerides, as well as the exogenous interfering substance, EDTA, on variant detection. Peripheral whole blood was collected from three patients with IRDs. Interfering substances were added to the blood samples, and DNA was extracted. The PrismGuide IRD Panel System detected 557 SNVs and 30 small indels in three patients with IRDs without adding potentially interfering substances, which was considered the control condition. Subsequently, the detection rates of these variants were determined in the presence of each potentially interfering substance. The variant-detection rates ranged from 99.6% to 99.8% for SNVs (*n* = 557) and were 100% for small indels (*n* = 30) (Fig 5A). In addition, the VAFs of the heterozygous and homozygous variants were close to the theoretical VAFs of 50% and 100%, respectively, and were comparable to those under the control condition (Fig 5B). These results showed that variant detection with the PrismGuide IRD Panel System was not affected by these potentially interfering substances at the concentrations tested.

**Fig 6:**
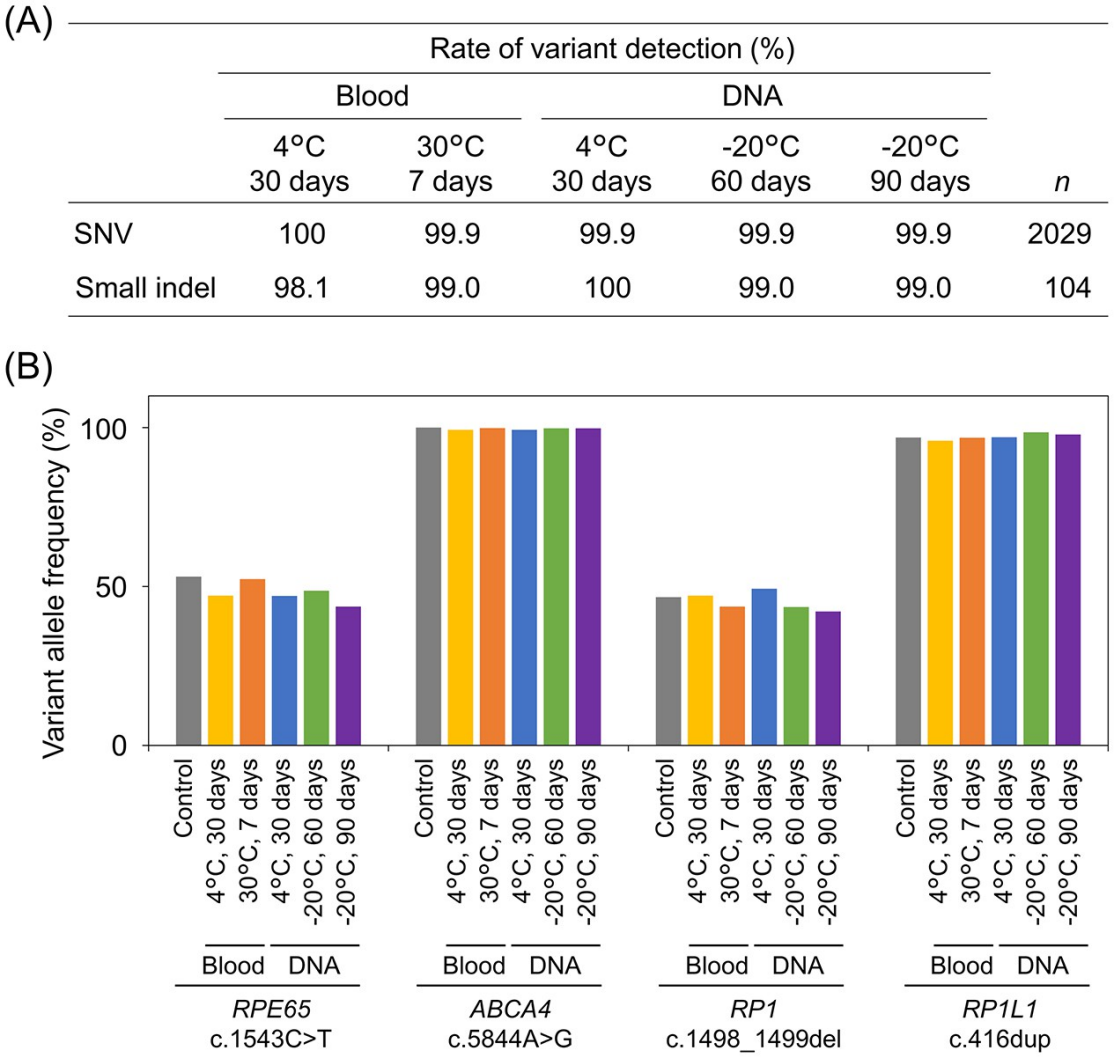
Stability of peripheral whole blood and genomic DNA samples for variant detection with the PrismGuide IRD Panel System. (A) The percentages of variant detection under the indicated storage conditions were shown. The percentage of variant detection was determined by comparing the number of variants detected in each storage condition in 11 clinical samples to that found in the control condition, where peripheral blood and DNA were immediately processed for library preparation without an extended storage period or unusual temperature conditions. Numbers of the variants are shown in parentheses. SNV, single-nucleotide variant; indel, insertion and deletion. (B) Variant allele frequencies of the representative variants observed in peripheral whole-blood and genomic-DNA samples after storage under the indicated conditions.

To evaluate variant detection under different storage conditions, peripheral whole blood collected from 11 patients with IRDs was examined. A part of the blood samples was stored at 4°C for 30 days and at 30°C for 7 days. Extracted DNA from the blood samples were stored under different storage conditions, including 4°C for 30 days, -20°C for 60 days, and -20°C for 90 days. The PrismGuide IRD Panel System identified 2,029 SNVs and 104 small indels from 11 patients with IRDs under control conditions, where the blood and DNA samples were not subjected to any storage condition. In the stored samples, the detection rates for the SNVs and small indels ranged from 98.1% to 100% (Fig 6A). Furthermore, the VAFs of the representative heterozygous and homozygous variants were close to the theoretical VAFs of 50% and 100%, respectively, and were comparable to those found under the control conditions (Fig 6B). These results indicated that variant detection using the PrismGuide IRD Panel System was not affected by these storage conditions.

## Discussion

### Clinical utility of the PrismGuide IRD Panel System

Comprehensive genomic profiling using targeted gene panels represents a powerful tool for identifying causative genes responsible for IRDs [12, 17, 27, 32, 41]. The advantages of a targeted gene panel over whole-exome and whole-genome sequencing are that it focuses on genomic regions having significant biological and clinical relevance, thereby providing a greater read depth for clinically important genes, reduced costs, less computational burden, and lower incidental findings [16]. In this study, we developed an 82-gene targeted gene panel, the PrismGuide IRD Panel System, to acquire genetic information regarding the causative genes responsible for IRDs. These 82 genes are known to be implicated in IRDs and serve as selective and predictive biomarkers for available gene therapy, enrollment in ongoing clinical trials, visual prognosis, and potential risk assessments for family members. In 2017, the U.S. Food and Drug Administration approved the *in vivo* gene therapy voretigene neparvovec-rzyl (Luxturna) for patients with biallelic variants in *RPE65*, which is included in the PrismGuide IRD Panel System (Table 1). Our accuracy study demonstrates that the PrismGuide IRD Panel System can detect four variants in *RPE65*, including NM_000329.3: c.1543C>T in the patient S815, NM_000329.3: c.1298A>G in S855, NM_000329.3: c133T>C and NM_000329.3: c.1543C>T in S960, in the accuracy study (Fig 2). Importantly, it detected two distinct *RPE65* variants from one patient (S960). Furthermore, this system correctly identifies the zygosity of these variants (Fig 2C). In addition, it is necessary for effective gene therapy to assess not only *RPE65* but also other genes related to IRDs to exclude that other genes are not causative for IRDs in a patient. Thus, we conclude that the PrismGuide IRD Panel System is useful for identifying patients eligible for voretigene neparvovec-rzyl (Luxturna) gene therapy.

Implementing voretigene neparvovec-rzyl (Luxturna) in clinical practice has paved the way for several ongoing gene therapies, including those that focus on several genes (including *RPE65*, *CEP290*, *PDE6B*, *RLBP1*, *USH2A*, *PDE6A*, *RHO*, *RPGR*, *CHM*, *RS1*, *MERTK*, *MYO7A*, *ABCA4*, *CNGA3*, *CNGB3*, and *PDE6B*), some of which are currently in phase 3 clinical trials [23–25, 42]. The PrismGuide IRD Panel System covers all these genes and, therefore, can help identify patients eligible for these ongoing clinical trials.

IRDs are high heterogeneous in both phenotypes and genotypes [1, 2]. Because of their heterogeneous nature and the overlapping of clinical phenotypes among the different types of IRDs, a definitive diagnosis is challenging. Macular dystrophies, including Stargardt disease and cone-rod dystrophy, comprise a large portion of IRD cases and would be difficult to distinguish from other types of IRDs at older ages. Thus, determining the causative gene is crucial for a definitive diagnosis in patients with IRD. For example, the identification of pathogenic variants in *ABCA4* can help diagnose Stargardt disease at older ages. As shown in Fig 2A and S2 Table, in the accuracy test, the PrismGuide IRD Panel System detected all three *ABCA4* variants tested in the patients S977 (NM_000350.3: c.1760+2T>G) and S1185 (NM_000350.3: c.4715C>T) with macular dystrophy in both cases and S1189 (NM_000350.3: c.880C>T) with cone-rod dystrophy. These results suggest that the PrismGuide IRD Panel System can reliably detect *ABCA4* variants and helps distinguish macular dystrophy and cone-rod dystrophy from other IRDs, in case the clinical diagnosis is not definitive.

Several reports have shown correlations between clinical phenotypes and pathogenic variants in genes, such as those in *RHO* and *ABCA4* [43, 44]. Therefore, the PrismGuide IRD Panel System can enhance the accuracy of prognosis concerning visual outcomes by identifying those pathogenic variants. Moreover, identifying causative variants will help determine inheritance patterns, thereby facilitating the prediction of the risks of genetic transmission within families.

### The PrismGuide IRD Panel System identifies SNVs and small indels accurately and reproducibly

The PrismGuide IRD Panel System effectively and consistently detected SNVs and small indels. The PrismGuide IRD Panel System showed 100% PPA and NPA for SNVs and small indels in the accuracy study (Fig 2A) and 100% PPA for repeatability and reproducibility in the precision study (Fig 3A). However, this study has some limitations. First, because IRDs are rare, a limited number of variants (35 SNVs and eight small indels from 43 patients with IRDs) was evaluated in the accuracy study. Second, the selected small indels were up to 17 base pairs long, leaving the ability of the PrismGuide IRD Panel System to detect larger indels unvalidated. Third, an extremely low or high GC content causes uneven coverage or even a lack of coverage across the genome in a sequencing-by-synthesis-based NGS platform employed with the PrismGuide IRD Panel System [35–37]. As variants in genomic regions with GC contents lower than 28% or higher than 68% were not assessed (Fig 2B), it is uncertain that the accuracy of variant detection in such genomic regions is comparable to that in other regions. The PrismGuide IRD Panel System includes *GUCY2D* and *NYX*, both of which have high GC contents in their coding regions. The first coding exon of *GUCY2D* (NM_000180.4) has a GC content of 76%, and pathogenic variants were found in this region in patients with IRDs [45]. In addition, *NYX* (NM_022567.2) has a GC content of 72% in the entire coding region [46]. Therefore, the accuracy of variant detection in these GC-rich regions may be lower than that in other regions. Despite these limitations, we consider that the highly accurate and reproducible PrismGuide IRD Panel System can still be implemented in clinical practice.

In contrast to the 100% PPA found for SNVs and small indels, the PrismGuide IRD Panel System failed to report one copy-number loss over exons 2 and 3 of *PED6B* in patient S952 (Table 2). The copy-number loss was confirmed using alternative methods, including PCR and whole-genome sequencing. To detect copy-number loss, the PrismGuide IRD Panel System determined the log_2_ ratio of the read depths for patients with IRDs compared with those for healthy individuals at three distinct genomic positions in both exons 2 and 3, which span 153 and 90 bases, respectively. Upon visual inspection of the sequencing reads of this genomic region using the Integrative Genomics Viewer, the log_2_ ratios at three genomic positions were -1.06, -1.12, and -1.04 in exon 2 and -1.03, -1.04, and -0.98 in exon 3, suggesting that the copy number likely decreased to one in this region. Given these observations, the PrismGuide IRD Panel System could have detected copy-number loss in patient S952 but failed to output the copy-number loss in the final report. Further investigations are underway to identify the reasons for this omission during the reporting process.

### The PrismGuide IRD Panel System is capable to detect *RPGR* ORF15 variants

The retina-specific, alternatively spliced form of *RPGR* contains ORF15 (NM_001034853.2: c.1754–c.3459) [39], which contains purine-rich and highly repetitive sequences, particularly in a 979-base pair segment in the central region (NM_001034853.2: c.2184-c.3162) [27–30]. This central region has low complexity, with 98.3% of the bases comprising purines, adenine or guanine. This composition results in a low read depth and poses challenges while using short-read and hybrid-capture NGS technology. Nevertheless, approximately 60% of disease-causing variants in *RPGR* are found in ORF15 [38, 39]. Therefore, it is crucial for the targeted gene panel to be capable of detecting ORF15 variants to avoid false-negative results. The PrismGuide IRD Panel System achieved a read depth above the variant call threshold throughout the ORF15 region and detected all five variants in the central region (NM_001034853.2: c.2184-c.3162; Fig 4A and 4C). Furthermore, the PrismGuide IRD Panel System provided VAFs close to the expected VAFs: 100% for hemizygous variants and 50% for heterozygous variants (Fig 4C). Thus, we conclude that the PrismGuide IRD Panel System could reliably detect ORF15 variants.

## Conclusion

Here, we present an analytical and clinical validation of a targeted gene panel, the PrismGuide IRD Panel System, designed for the comprehensive genome profiling of 82 genes that are clinically relevant to IRDs. Given its highly accurate and reproducible variant-detection performance and regulatory approval from the Ministry of Health, Labor, and Welfare in Japan, we believe that the PrismGuide IRD Panel System can be applied in clinical practice to support definitive IRD diagnosis, selection of patients eligible for gene therapy, enrollment in ongoing clinical trials, prediction of visual prognosis, and assessments of potential risks for family members.

## Supporting information

S1 Table List of representative variants in human reference genome, NA24385. (DOCX)

S2 Table Patient characteristics and the list of variants analyzed to determine the accuracy for the detection of SNVs, indels and copy-number loss. (DOCX)

S3 Table List of genes and their RefSeq ID defined in the PrismGuide IRD Panel System. (DOCX)
