# Supplementary material for: Clinical and analytical validation of an 82-gene comprehensive genome-profiling panel for identifying and interpreting variants responsible for inherited retinal dystrophies
Source: PLoS One. 2024 Jun 13;19(6):e0305422. doi: 10.1371/journal.pone.0305422 (PMC11175448; doi:10.1371/journal.pone.0305422)
Supplement: S1 Table — (DOCX) [file pone.0305422.s001.docx]

**Supporting information**

**S1 Table. List of representative variants in human reference genome, NA24385**

| Gene | Chromosome | Genome position^#1^ | | Ref. ^#2^ | Alt.^#3^ | Variant  type | Zygosity^#4^ |
| --- | --- | --- | --- | --- | --- | --- | --- |
|  |  | Start | End |  |  |  |  |
| *USH2A* | 1 | 215674614 | 215674614 | C | A | SNV | Hetero. |
| *USH2A* | 1 | 216046439 | 216046439 | A | G | SNV | Hetero. |
| *USH2A* | 1 | 216289320 | 216289320 | T | A | SNV | Hetero. |
| *USH2A* | 1 | 216323590 | 216323590 | C | G | SNV | Hetero. |
| *ABCA4* | 1 | 93996161 | 93996161 | C | A | SNV | Hetero. |
| *ABCA4* | 1 | 94098927 | 94098927 | C | T | SNV | Hetero. |
| *RPGRIP1* | 14 | 21301034 | 21301034 | C | A | SNV | Hetero. |
| *RPGRIP1* | 14 | 21302571 | 21302571 | A | G | SNV | Homo. |
| *RP1L1* | 8 | 10608972 | 10608972 | G | A | SNV | Hetero. |
| *ABCA4* | 1 | 93996198 | 93996198 | A | G | SNV | Hetero. |
| *RHO* | 3 | 129532420 | 129532420 | C | T | SNV | Hetero. |
| *ADGRV1* | 5 | 90756446 | 90756447 | T | TC | Indel | Hetero. |
| *EYS* | 6 | 64307084 | 64307086 | TAG | T | Indel | Hetero. |
| *MYO7A* | 11 | 77184725 | 77184747 | GGGAGGCGGGG  ACACCAGGGCCT | G | Indel | Hetero. |
| *POC1B* | 12 | 89472277 | 89472287 | TAGAAAGAAGA | T | Indel | Hetero. |

^#1^The genome position is indicated according to the human reference genome, GRCh38.

^#2^Reference variant in the human reference genome, GRCh38

^#3^Alternate variant in NA24385

^#4^Hetero, heterozygote; Homo., homozygote
