# Supplementary material for: Clinical and analytical validation of an 82-gene comprehensive genome-profiling panel for identifying and interpreting variants responsible for inherited retinal dystrophies
Source: PLoS One. 2024 Jun 13;19(6):e0305422. doi: 10.1371/journal.pone.0305422 (PMC11175448; doi:10.1371/journal.pone.0305422)
Supplement: S2 Table — (DOCX) [file pone.0305422.s002.docx]

**Supporting information**

**S2 Table. Patient characteristics and the list of variants analyzed to determine the accuracy for the detection of SNVs, indels and copy-number loss.**

| Sample ID | Disease | Age | Gender | Gene | Variant | Variant type | Zygosity of the variant |
| --- | --- | --- | --- | --- | --- | --- | --- |
| S815 | LCA | 42 | M | *RPE65* | c.1543C>T | SNV | Homo. |
| S855 | RP | 80 | F | *RPE65* | c.1298A>G | SNV | Hetero. |
| S884 | RP | 66 | M | *RP1L1* | Deletion of exon 3-4 | CNV-loss | Hetero. |
| S888 | RP | 74 | F | *RDH12* | c.920G>A | SNV | Homo |
| S952 | RP | 50 | F | *PDE6B* | Deletion of exon 2-3 |  | Hetero. |
| S960 | LCA | 45 | M | *RPE65* | c.133T>C | SNV | Hetero. |
|  |  |  |  |  | c.1543C>T | SNV | Hetero. |
| S975 | BCR | 46 | F | *CYP4V2* | c.802-8_810delinsGC | Indel | Homo |
| S977 | MD | 30 | F | *ABCA4* | c.1760+2T>G | SNV | Hetero. |
| S983 | RP | 48 | F | *EYS* | Deletion of exon 6-8 | CNV-loss | Hetero. |
| S988 | RP | 49 | F | *USH2A* | c.8559-2A>G | SNV | Homo |
| S994 | RP | 61 | F | *RP1* | c.1186C>T | SNV | Hetero. |
| S1003 | RP | 32 | F | *PRPF31* | c.527+1G>A | SNV | Hetero. |
| S1017 | RP | 48 | F | *RP1* | c.5797C>T | SNV | Hetero. |
| S1031 | RP | 48 | M | *ADGRV1* | c.2309A>G | SNV | Hetero. |
| S1042 | RP | 48 | F | *PRPF31* | Deletion of all exons | CNV-loss | Hetero. |
| S1061 | RP | 48 | M | *RPGR* | c.3092del | Indel | Homo |
| S1073 | RP | 33 | F | *MERTK* | c.225del | Indel | Homo |
| S1094 | RP | 33 | M | *RPGR* | c.247G>A | SNV | Homo |
| S1106 | RP | 56 | F | *RDH12* | c.437T>A | SNV | Homo |
| S1119 | RP | 29 | M | *CNGB1* | c.2225del | Indel | Homo |
| S1134 | RS | 40 | M | *RS1* | c.638G>A | SNV | Homo. |
| S1140 | n/a^#1^ | 77 | M | *PDE6B* | c.1669C>T | SNV | Hetero. |
| S1144 | US | 31 | F | *MYO7A* | c.6106C>T | SNV | Homo. |
| S1145 | RP | 53 | F | *PRPF31* | Deletion of exons2-3 | CNV-loss | Hetero. |
| S1146 | RP | 30 | F | *PROM1* | c.2464A>G | SNV | Hetero. |
| S1147 | RP | 58 | F | *NR2E3* | c.215A>G | SNV | Hetero. |
| S1148 | RP | 37 | F | *CEP290* | c.1078C>T | SNV | Hetero. |
| S1159 | RP | 69 | M | *RHO* | c.50C>T | SNV | Hetero. |
| S1165 | RP | 63 | M | *EYS* | c.2528G>A | SNV | Homo. |
| S1182 | RP | 51 | F | *EYS* | c.4957dup | Indel | Homo. |
| S1185 | MD | 57 | F | *ABCA4* | c.4715C>T | SNV | Hetero. |
| S1188 | RP | 76 | F | *CYP4V2* | c.1020G>A | SNV | Homo. |
| S1189 | CRD | 62 | M | *ABCA4* | c.880C>T | SNV | Homo. |
| S1196 | RP | 30 | M | *RP2* | c.331A>C | SNV | Homo. |
| S1197 | RP | 72 | M | *EYS* | c.6563T>C | SNV | Hetero. |
| S1201 | RP | 29 | F | *RHO* | c.1040C>T | SNV | Hetero. |
| S1203 | RP | 34 | F | *USH2A* | c.2802T>G | SNV | Hetero. |
| S1205 | CRD | 48 | F | *BEST1* | c.1037C>A | SNV | Hetero. |
| S1210 | RP | 61 | F | *USH2A* | c.12168G>A | SNV | Hetero. |
| S1226 | RP | 29 | M | *IMPDH1* | c.1079C>T | SNV | Hetero. |
| S1233 | RP | 52 | F | *GUCY2D* | c.1871G>A | SNV | Hetero. |
| S1235 | RP | 43 | F | *CNGA1* | c.1417del | Indel | Homo. |
| S1251 | CHM | 59 | M | *CHM* | c.757C>T | SNV | Homo. |
| S1275 | RP | 47 | M | *RPGR* | c.2426_2427del | Indel | Homo. |
| S1276 | RP | 55 | M | *CNGA1* | c.253del | Indel | Hetero. |
| S1319 | RP | 40 | M | *TULP1* | c.1145T>C | SNV | Homo. |
| S1322 | RP | 63 | F | *PRPH2* | c.556G>A | SNV | Hetero. |

The indicated variants and their zygosity were determined by the alternative targeted gene panel and Sanger sequencing.

The age at which blood sample was obtained is indicated.

BCR, Bietti crystalline retinopathy; CHM, Choroideremia; CRD, Cone-rod dystrophy; Hetero, heterozygote; Homo, homozygote; LCA, Leber congenital amaurosis; MD, Macular dystrophy; RP, Retinitis pigmentosa; RS, Retinoschisis; US, Usher syndrome.

^#1^Parent of a proband with RP
