# Supplementary material for: Clinical and analytical validation of an 82-gene comprehensive genome-profiling panel for identifying and interpreting variants responsible for inherited retinal dystrophies
Source: PLoS One. 2024 Jun 13;19(6):e0305422. doi: 10.1371/journal.pone.0305422 (PMC11175448; doi:10.1371/journal.pone.0305422)
Supplement: S3 Table — (DOCX) [file pone.0305422.s003.docx]

**Supporting information**

**S3 Table. List of genes and their RefSeq ID defined in the PrismGuide IRD Panel System**

| Gene | RefSeq ID |
| --- | --- |
| *ABCA4* | NM_000350.3 |
| *ADGRV1* | NM_032119.4 |
| *AIPL1* | NM_014336.5 |
| *BEST1* | NM_004183.4 |
| *C8orf37* | NM_177965.4 |
| *CA4* | NM_000717.5 |
| *CACNA1F* | NM_005183.4 |
| *CDH23* | NM_022124.6 |
| *CDHR1* | NM_033100.4 |
| *CEP290* | NM_025114.4 |
| *CERKL* | NM_001030311.3 |
| *CFAP410* | NM_004928.3 |
| *CHM* | NM_000390.4 |
| *CLRN1* | NM_174878.3 |
| *CNGA1* | NM_000087.5 |
| *CNGA3* | NM_001298.3 |
| *CNGB1* | NM_001297.5 |
| *CNGB3* | NM_019098.5 |
| *CRB1* | NM_201253.3 |
| *CRX* | NM_000554.6 |
| *CYP4V2* | NM_207352.4 |
| *DHDDS* | NM_024887.4 |
| *DRAM2* | NM_178454.6 |
| *EYS* | NM_001292009.2, NM_001142800.2 |
| *FAM161A* | NM_001201543.2 |
| *FSCN2* | NM_001077182.3 |
| *GNAT2* | NM_005272.5 |
| *GRK1* | NM_002929.3 |
| *GUCA1A*  *GUCY2D*  *IDH3B*  *IMPDH1*  *IMPG2*  *IQCB1*  *KCNV2*  *KLHL7*  *LRAT*  *MAK*  *MERTK*  *MYO7A*  *NMNAT1*  *NR2E3*  *NRL*  *NYX*  *PCARE*  *PDE6A*  *PDE6B*  *PDE6C*  *PDE6G*  *POC1B*  *PRCD*  *PROM1*  *PRPF3*  *PRPF31*  *PRPF6*  *PRPF8*  *PRPH2*  *RBP3*  *RDH12*  *RDH5*  *RGR*  *RGS9BP*  *RHO*  *RLBP1*  *ROM1*  *RP1*  *RP1L1*  *RP2*  *RP9*  *RPE65*  *RPGR*  *RPGRIP1*  *RS1*  *SAG*  *SEMA4A*  *SNRNP200*  *SPATA7*  *TOPORS*  *TTC8*  *TULP1* | NM_000409.5  NM_000180.4  NM_006899.5  NM_000883.4  NM_016247.4  NM_001023570.4  NM_133497.4  NM_001031710.3  NM_004744.5  NM_001242957.3  NM_006343.3  NM_000260.4  NM_022787.4  NM_014249.4  NM_006177.5  NM_022567.2  NM_001029883.3  NM_000440.3  NM_000283.4  NM_006204.4  NM_002602.4  NM_172240.3  NM_001077620.3  NM_006017.3  NM_004698.4  NM_015629.4  NM_012469.4  NM_006445.4  NM_000322.5  NM_002900.3  NM_152443.3  NM_002905.5  NM_001012720.2  NM_207391.3  NM_000539.3  NM_000326.5  NM_000327.4  NM_006269.2  NM_178857.6  NM_006915.3  NM_203288.2  NM_000329.3  NM_001034853.2, NM_000328.3  NM_020366.4  NM_000330.4  NM_000541.5  NM_022367.4  NM_014014.5  NM_018418.5  NM_005802.5  NM_198309.3  NM_003322.6 |
| *USH2A* | NM_206933.4 |
| *ZNF513* | NM_144631.6 |
